# New Growths of Large Intestine

**Published:** 1915-07

**Authors:** 


**New Growths of Large Intestine.** T. Copeland Savage, Auckland, N. Zealand Med. Jour., Feb. 1915. In 10 years' practice, the following cases were noted:

| | |
|---|---|
| In the caecum or ileo-caecal region ... | 5 |
| " " ascending colon ... ... ... | 3 |
| " " hepatic flexure ... ... ... | 4 |
| " " transverse colon ... ... ... | 3 |
| " " Splenic flexure ... ... ... | 1 |
| " " descending colon ... ... | 3 |
| " " sigmoid ... ... ... | 20 |
| " " pelvic colon ... ... ... | 8 |
| | 47 |

Thus, of the 47 cases, 28 or 68 per cent, were at or below the point at which the bowel crosses the left pelvic brim and not easily accessible for examination either by the abdominal or rectal route.

Sex.—28 were in men and 19 in women.

Age.—Under 40, 8 cases, of which the youngest was 31, and two were 32.

| | |
|---|---|
| Between 40 and 50 ... ... | 12 cases |
| Between 50 and 60 ... ... | 10 cases |
| Over 60 ... ... ... | 17 cases |

Æteology.—I am unable to offer any suggestions as to the special aetiological factors.

Nature of the Growth.—All except three have been carcinomatous ulcers, occupying in most cases the entire circumference of the bowel, but in two I was lucky enough to get the case when the ulcer had not advanced to such a degree.

Three cases were polypoid tumours, of which two occurring at the ileo-caecal orifice or within an inch of it, were simple pedunculated fibromata and after a more or less prolonged history of attacks the pain, with incomplete obstruction, finally culminated by causing intussusception. The third is a remarkable case (I have here the specimen) of a carcinomatous polyp of the transverse colon, which also culminated in intussusception.

